# Genome-wide association study of population-standardised cognitive performance phenotypes in a rural South African community

**DOI:** 10.1038/s42003-023-04636-1

**Published:** 2023-03-27

**Authors:** Cassandra C. Soo, Jean-Tristan Brandenburg, Almut Nebel, Stephen Tollman, Lisa Berkman, Michèle Ramsay, Ananyo Choudhury

**Affiliations:** 1 Sydney Brenner Institute for Molecular Bioscience, Faculty of Health Sciences, University of the Witwatersrand, Johannesburg, South Africa; 2 Division of Human Genetics, National Health Laboratory Service and School of Pathology, Faculty of Health Sciences, University of the Witwatersrand, Johannesburg, South Africa; 3 Institute of Clinical Molecular Biology, Kiel University, 24105 Kiel, Germany; 4 MRC/Wits Rural Public Health and Health Transitions Research Unit, School of Public Health, Faculty of Health Sciences, University of the Witwatersrand, Johannesburg, South Africa; 5 Department of Social and Behavioral Sciences, Harvard T.H. Chan School of Public Health, Boston, MA USA; 6 Harvard Center for Population and Development Studies, Harvard University, Cambridge, MA USA

**Keywords:** Genome-wide association studies, Behavioural genetics

## Abstract

Cognitive function is an indicator for global physical and mental health, and cognitive impairment has been associated with poorer life outcomes and earlier mortality. A standard cognition test, adapted to a rural-dwelling African community, and the Oxford Cognition Screen-Plus were used to capture cognitive performance as five continuous traits (total cognition score, verbal episodic memory, executive function, language, and visuospatial ability) for 2,246 adults in this population of South Africans. A novel common variant, rs73485231, reached genome-wide significance for association with episodic memory using data for ~14 million markers imputed from the H3Africa genotyping array data. Window-based replication of previously implicated variants and regions of interest support the discovery of African-specific associated variants despite the small population size and low allele frequency. This African genome-wide association study identifies suggestive associations with general cognition and domain-specific cognitive pathways and lays the groundwork for further genomic studies on cognition in Africa.

## Abstract (web summary)

A genome-wide association study for five cognitive phenotypes in a rural South African community suggests several variants associated with general cognition and domain-specific cognitive pathways in this cohort.

## Introduction

Normal cognitive function is an essential determinant for health and quality of life indicators. Evolutionary evidence suggests that along with increased cranial complexity, humans developed complex communication, abstract thought, and reasoning through their increased capacity for social learning^1^. Genome-wide association studies (GWAS) for cognitive function have been challenging despite twin studies suggesting heritability scores up to ~80% for various cognitive phenotypes^1–10^. The question of heritability is further complicated by evidence that it varies across the lifespan and has different trajectories throughout the life course, with relative stability observed from middle to old age^4,9–13^. Despite the complex, polygenic, and pleiotropic nature of neurocognitive phenotypes, meta-analyses with larger sample sizes (>50,000) were able to detect associations with single nucleotide polymorphisms (SNPs) and have successfully replicated findings with genome-wide significance (*p* < 5 × 10^−8^)^3,6,14–17^. In order to perform these meta-analyses, general cognitive ability (or Spearman’s *g*) was derived from diverse positively but not perfectly correlated cognitive performance tests (capturing ~40% of phenotypic variance), or proxy phenotypes such as educational attainment^3,6,9,14–18^. Studies have used different metrics, measures, and tests to describe traits such as intelligence (fluid or crystallised), general cognitive function, and domain-specific cognitive outcomes hence the adoption of *g* to account for testing heterogeneity^2–4,9,18–20^. Functional studies have shown that each of the cognitive domains has an impact on gene expression in different regions of the brain, making latent cognitive ability an amalgamation of activity within the brain acting through different biological pathways^4,9,21–29^. A further limitation of these studies is that they suffer from sample heterogeneity in terms of the age of participants, socio-economic status (SES), and participant’s access to education^3,12,15,20^. As noted, cognitive trajectory changes throughout lifespan require participants to be within similar age ranges to accurately capture cognitive ability for comparative studies^4,11–13^. Education is also a major moderating factor for assessing cognitive ability, with evidence suggesting that genes associated with educational attainment are an artefact of positive selection^1,9^. Cognitive performance tests typically rely on literacy and numeracy, which is a source of bias in many low-income populations^2,4,9,13,18,20,30,31^. In some settings, SES is a major determinant influencing access to education, so cognitive batteries may be measuring educational exposure rather than innate cognitive function^2,4,13,18,20,30,31^.

There is little research on the genetics of cognitive function in African populations, or in those of African ancestry^4,5,32–34^. The lack of diverse ethnic representation in studies to date limits the discovery of associated variants as differences in linkage disequilibrium (LD) (with generally smaller LD blocks in Africans compared to Europeans), could enhance the discovery of causal variants in African populations^35^. The Health and Aging in Africa: A Longitudinal Study of an INDEPTH Community in South Africa (HAALSI) collected baseline cognition data for over 5000 older adults in Bushbuckridge, rural Mpumalanga, South Africa (SA)^36^. A sub-set of 2246 participants from this study were also recruited as part of the Africa Wits-INDEPTH Partnership for Genomic Studies (AWI-Gen) for whom genotype data were available from the Illumina Human Heredity and Health in Africa (H3Africa) array^37,38^. The combined dataset with phenotype and genotype data was used to explore genetic associations with latent cognitive ability based on multiple quantitative traits (total cognition score, verbal episodic memory, executive function, language, and visuospatial ability) for ~2000 individuals in five independent GWAS using LD structure specific to those of African ancestry in SA. To the best of our knowledge, this is the first large study in Southern Africa to explore genetic contributions to non-pathological cognitive performance.

## Results

### Genome-wide association study results

We performed GWAS for five cognitive traits (Table 1). This sample had more women (~58%) than men. The participants had little access to education, where ~77% of the sample population had not progressed beyond primary school. After rank normalisation, cognitive domain data were available for 1887 genotyped participants. The ranges displayed in Table 1 are for the population-standardised *z*-scores and show a particularly wide range of performance for visuospatial cognition. Total cognition score data were available for all 2211 genotyped participants. The imputed dataset included 13,972,012 SNPs.

**Table 1:** Descriptive statistics showing cognitive trait and covariate distribution.

Measure	*n*	Range (Median[IQR])	Mean or percentage	SD
Age (years)	2211	40 to 80 (57 [49–67])	57.69	10.93
Sex	2211			
Female			58.53% (1294)	
Male			41.47% (917)	
Level of education	2211			
No formal education			39.85% (881)	
Primary			37.63% (832)	
Secondary			18.72% (414)	
Tertiary			3.80% (84)	
Cognitive measures				
Total cognition score	2211	0–24 (12 [9–14])	11.68	4.38
Cognitive domains^a^	1887			
Executive function		−4.69 to 4.33 (−0.14 [−1.32–1.30])		
Episodic memory		−3.91 to 1.46 (0.29 [−0.54–0.79])		
Language		−2.53 to 1.28 (0.13 [−0.41–0.51])		
Visuospatial cognition		−10.63 to 4.77 (0.49 [−1.75–2.33])		

^a^Cognitive domain data given as *z*-scores prior to rank normalisation with median and interquartile ranges rounded up to two decimal places.

**Fig. 1:**
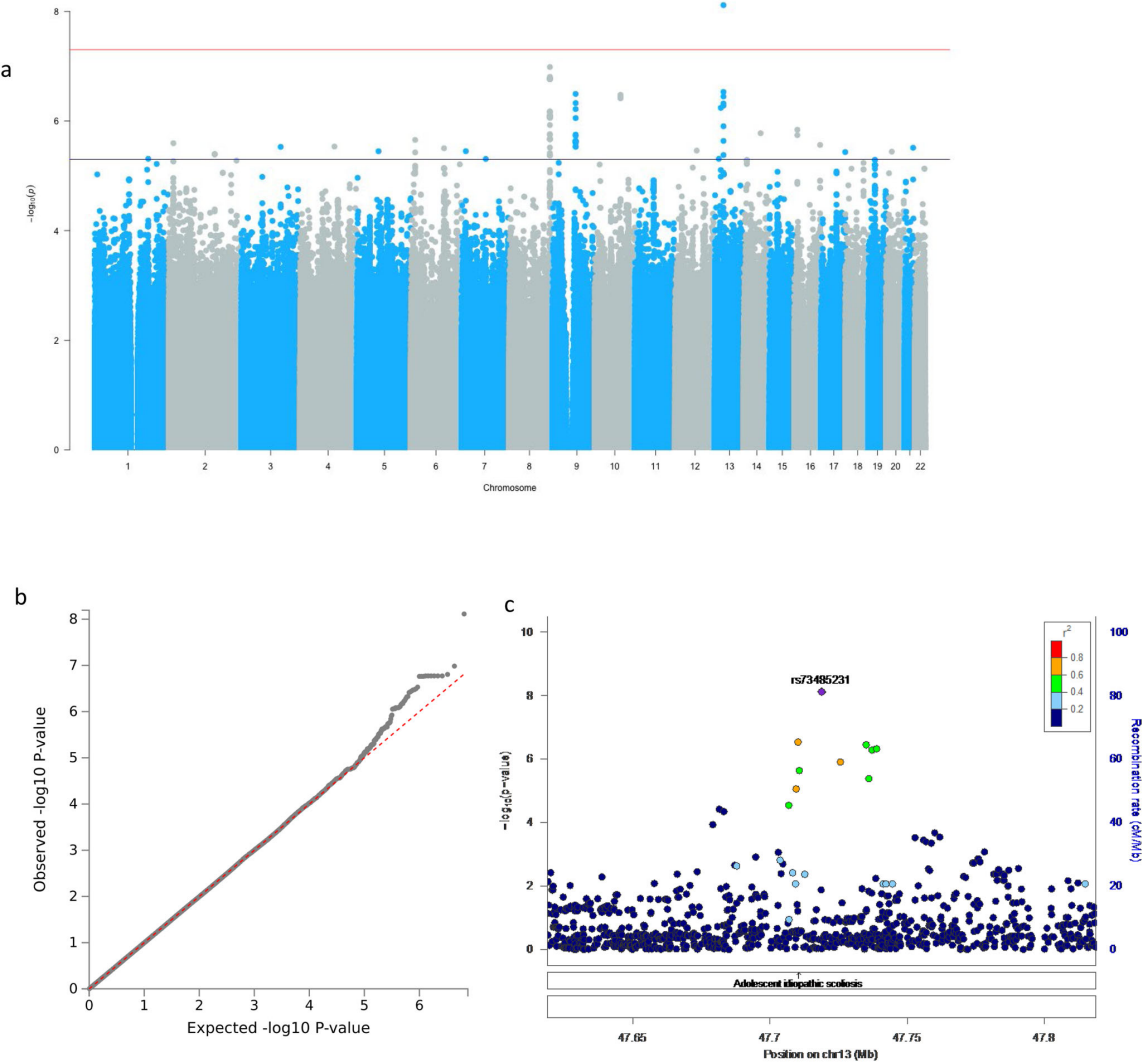
Genome-wide and suggestive associations with verbal episodic memory. **a** Manhattan plot. Genome-wide significance cut-off 5 × 10^−8^ is shown by red line and suggestive cut-off 5 × 10^−6^ is shown by blue line. **b** QQ-plot (*λ* = 0.99) for association summary statistics **c** Locus zoom plot for rs73485231. LD is based on a South African LD panel.

The GWAS for verbal episodic memory, identified a genome-wide significant signal for rs73485231 (*p* = 7.70 × 10^−9^, β = 0.24, SE = 0.04) on chromosome 13 (Fig. 1a shows the Manhattan plot, b the QQ-plot with *λ* = 0.99, c and the Locus zoom plot). The mean episodic memory score was significantly lower in G homozygotes (*p* = 5.4 × 10^−4^) (Supplementary Fig. 1a). This intergenic SNP between *GNG5P5* and *HTR2A* had a notably higher minor allele frequency (MAF) in Africans (AFR) (MAF = 0.13), compared to Europeans (EUR), Americans (AMR) and Asians (EAS and SAS) 1000 G Project super population groups (Table 2). Although no previous associations with cognition had been reported, GWAS Catalog reported this SNP to be associated with adolescent idiopathic scoliosis (Fig. 1c). A suggestive signal, rs140372794 (*p* = 1.04 × 10^−7^, β = 0.33, SE = 0.06), was observed on chromosome 8 (Supplementary Data 1). This SNP, along with a second suggestive variant (rs62529410, *p* = 7.02 × 10^−7^) within 27 kb of it, falls within 100 kb of *LINC02055*, a long intergenic non-protein coding RNA gene harbouring several SNPs previously associated with mathematical ability and general cognitive function. Gene-based association (Supplementary Table 1) yielded two suggestive gene signals; one for *TRPM6* on chromosome 9 (minimum *p* = 2.31 × 10^−6^), which encodes a magnesium channel protein^39,40^, and another for *BACE2* on chromosome 21 (minimum *p* = 3.08 × 10^−6^) which codes for an essential enzyme for the cleavage of *β*-Amyloid and the development of Alzheimer’s disease (AD)^41–43^.

**Table 2:** Minor allele frequency distribution of reported association signals in our sample compared to 1000 genomes super populations.

Trait	Variant ID	Minor allele	MAF this study	MAF AFR	MAF AMR	MAF EAS	MAF EUR	MAF SAS
Memory	rs73485231	A	0.14	0.13	0.02	0.02	0.02	0.07
Memory	rs140372794	A	0.05	0.04	0.01	0	0	0
Language	rs140578927	C	0.01	0.01	0	0	0	0
Executive function	rs3845674	T	0.23	0.23	0.30	0.40	0.25	0.20
Visuospatial ability	rs191611493	T	0.03	0.03	0.00	0	0	0
Total cognition score	rs138832740	C	0.03	0.04	0	0	0	0

1000 Genomes Project Super Populations groups AFR (African), AMR (Admixed American), EAS (East Asian), EUR (European), and SAS (South Asian).

**Fig. 2:**
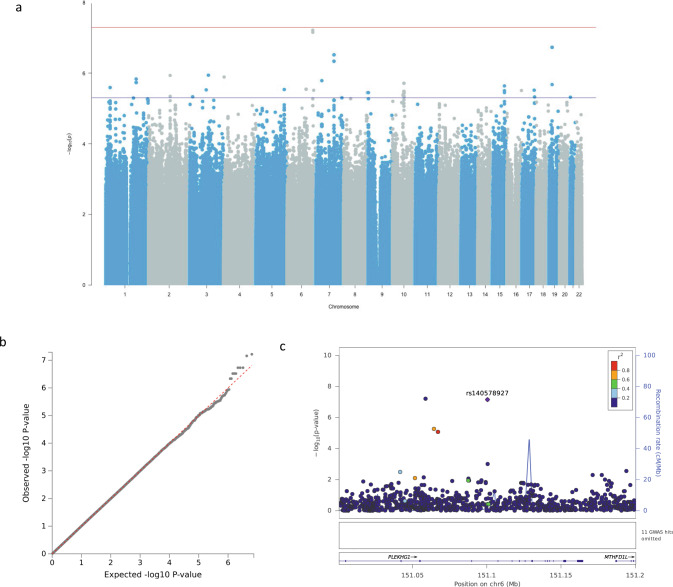
Genome-wide and suggestive associations with language. **a** Manhattan plot. Genome-wide significance cut-off 5 × 10^−8^ is shown by a red line, and suggestive cut-off 5 × 10^−6^ is shown by a blue line. **b** QQ-plot (*λ* = 1.00) for association summary statistics. **c** Locus zoom plot for rs140578927. LD values are based on a South African LD panel.

Our GWAS for language detected a near genome-wide significant association on chromosome 6 (rs140578927, *p* = 6.99 × 10^−8^, β = 0.65) (Fig. 2a, b). The MAF (C allele) for rs140578927 was 0.01 in our cohort and had not been reported in population groups other than the African supergroup in the 1000 G Project (Table 2). Despite its rarity, heterozygous individuals had higher mean language performance scores than homozygous individuals (Supplementary Fig. 1b). FUMA output indicated the nearest gene to be *PLEKGH1* which has been associated with blood pressure, white matter intensity, and cortical volume^44–46^. Regional lookup places it downstream of *MTHFD1L*, which had been associated with late-onset Alzheimer’s disease and coronary artery disease^47,48^. A series of suggestive signals associated with language in this cohort are listed in Supplementary Table 1. Gene-based output (Supplementary Table 1) suggested two genes encoding mitochondrial proteins on chromosome 15; *MRPL46* associated with depressive disorders (minimum *p* = 6.24 × 10^−5^)^49,50^ and *MRPS11* (minimum *p* = 3.16 × 10^−6^) linked to body-mass index (BMI)^51^.

**Fig. 3:**
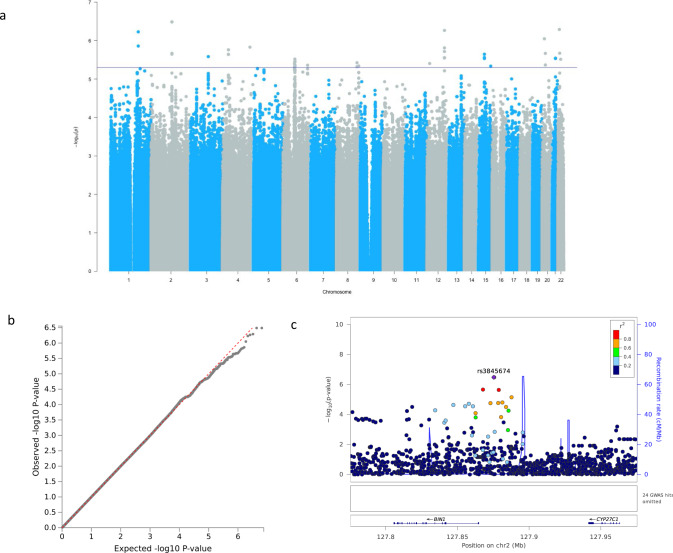
Genome-wide and suggestive associations with executive function. **a** Manhattan plot. No genome-wide or near genome-wide significant signals. Suggestive cut-off 5 × 10^−6^ shown by a blue line. **b** QQ-plot (*λ* = 1.00) for association summary statistics. **c** Locus zoom plot for rs3845674. LD values are based on a South African LD panel.

Genome-wide analysis results for executive function yielded only suggestive signals (Fig. 3a and Supplementary Data 1); however, rs3845674 is of particular interest due to its proximity to *BIN1* (Fig. 3c). This gene has been reported in multiple AD studies^52–54^. The effect allele of rs3845674 (G) has an allele frequency of ~77% in our sample, and homozygous carriers of this allele had significantly reduced executive function compared to heterozygous and homozygous T individuals (Supplementary Fig. 1c).

**Fig. 4:**
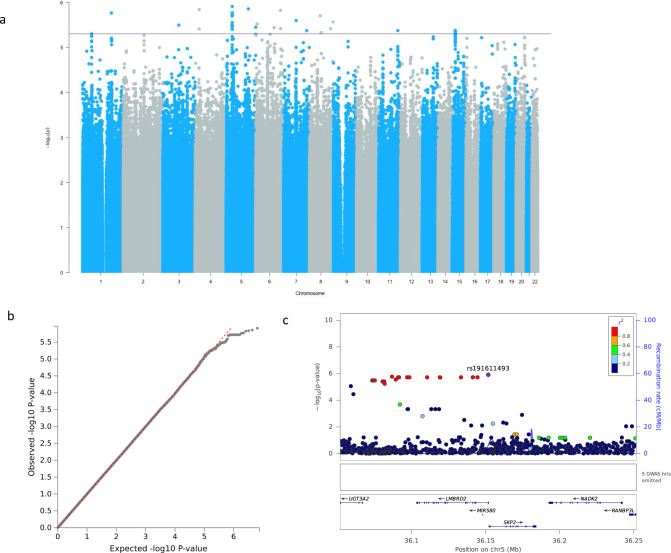
Genome-wide and suggestive associations with visuospatial ability. **a** Manhattan plot. No genome-wide or near genome-wide significant signals. Suggestive cut-off 5 × 10^−6^ shown by a blue line. **b** QQ-plot (*λ* = 1.00) for association summary statistics. **c** Locus zoom plot for rs191611493. LD values are based on a South African LD panel.

No genome-wide associations were observed for visuospatial ability (Fig. 4a, b), but a series of suggestive signals in LD falling within the gene *LMBRD2* are shown in Fig. 4c represented by rs191611493 which had the lowest *p* value (*p* = 1.23 × 10^−6^, β = 0.39, SE = 0.08) (Fig. 4a, b). The frequency of the effect allele was very low (Supplementary Data 1) and it did not have a significant effect on performance in this cohort (Supplementary Fig. 1d). Along with *LMBRD2*, gene-based analysis results implicated *DHX15, TRPC7*, *DTX2*, *UPK3B* and *POMZP3* (Supplementary Table 1).

**Fig. 5:**
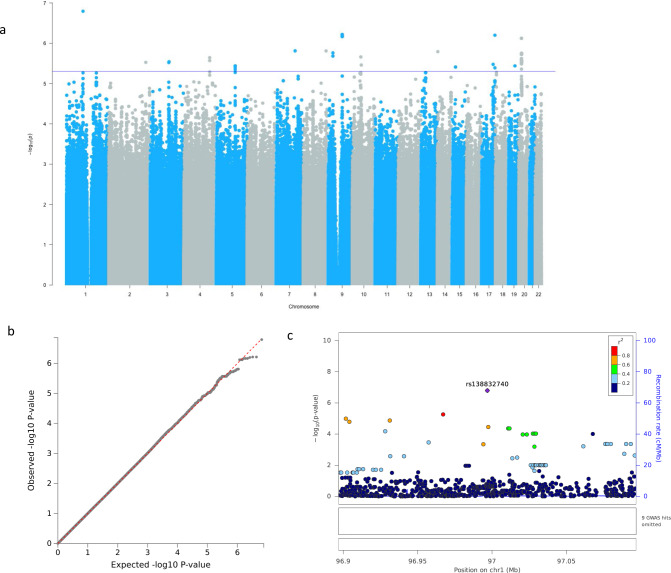
Genome-wide and suggestive associations for total cognition score. **a** Manhattan plot. No genome-wide or near genome-wide significant signals. Suggestive cut-off 5 × 10^−6^ shown by a blue line. **b** QQ-plot (*λ* = 1.00) for association summary statistics. **c** Locus zoom plot for rs138832740. LD values are based on a South African LD panel.

Although no SNPs reached genome-wide significance for association with the total cognition score (Fig. 5a, QQ-plot 5b, and Supplementary Data 1), the lead SNP rs138832740 (*p* = 1.61 × 10^−7^, β = −2.01, SE = 0.38) was African ancestry-specific according to the 1000 G Project dataset (Table 2). No previous associations had been reported for rs138832740, likely due to its low frequency and apparent continental specificity. The closest gene to this SNP is *RN7SL831P* which has been reported in behavioural traits and BMI. Only one participant was homozygous for the C allele, but a significant difference (*p* = 2.4 × 10^−3^) between performance was observed between heterozygous individuals and those who were homozygous for the major allele (Supplementary Fig. 1e). Two genes (*RBFOX3* and *MACROD2)*, although they did not meet gene-wide significance, code for proteins which are highly expressed in the central nervous system and integral to neuron development (Supplementary Table 1).

### GWAS replication

Exact replication of previously reported genome-wide significant variants associated with various cognitive function phenotypes was not achieved; however, using window-based methods proved to be sufficient to represent replication of our signals in other studies. The top observed association signals for each cognitive trait (Supplementary Data 1) were used for our window-based replication analysis. We reported replication of previously reported genome-wide significant SNPs (marked with an asterisk in Supplementary Data 2) and suggestive signals for memory and total cognition score.

The lowest *p* value observed for episodic memory window-based replication was for rs10773290 (*p* = 3.68 × 10^−4^), which was previously reported by ref. ^55^ as a suggestive signal for working memory along with two other markers. For rs8067235 (our study *p* = 4.55 × 10^−4^), a near genome-wide significant signal (*p* = 6.00 × 10^−8^) was observed by ref. ^56^ for association with memory performance.

For the total cognition score, we reported all window-based replication signals with *p* < 5 × 10^−4^ in Supplementary Data 2. Using the cut-off of 5 × 10^−3^, we managed to exactly replicate two suggestive signals: one for cognitive performance (rs2616984, *p* = 1.86 × 10^−3^), which also fell below our window-based replication threshold (*p* = 1.44 × 10^−4^), and one for general cognitive ability (rs1512144, *p* = 1.11 × 10^−3^ and window *p* = 2.81 × 10^−4^). Through widow-based replication, we further replicated 14 signals that had reached genome-wide significance in their respective studies for the traits of general cognitive ability and cognitive function. A further 40 SNPs were replicated for previously reported suggestive signals for the traits, cognitive performance, and generalised correlation coefficient along with the other traits mentioned above.

For the rest of the remaining cognitive traits; language, executive function, and visuospatial cognition, we failed to replicate previously reported suggestive signals within our cut-off threshold. These are presented in Supplementary Data 2.

## Discussion

Few genetic association studies for cognitive traits have been performed in continental Africans and meta-analyses suffer from the limitations of grouping different cognitive phenotypes together, of which data was collected using different screening tools^4,57^. Although a number of recent epidemiological studies assessing cognitive function and various associated phenotypes have been published, there is still a dearth of genomic data available from Africa.

Traditional cognition batteries are often ill-adapted to screening populations with lower literacy and numeracy levels, confounding comparative analyses^4,57^. This is especially evident in settings where educational attainment is strongly influenced by SES^1,4,9,57^. Adaptations of the standard mini-mental state examination (MMSE) to screen for cognitive impairment linked to ageing, and neurological and psychiatric conditions have been used since its inception as a simple way to assess cognitive traits such as orientation, comprehension, language, memory, and tasks for reading, writing, and drawing^58^. The main limitation of the MMSE is that it cannot be administered to individuals who are illiterate, making it unsuitable for capturing cognitive function data in communities with low literacy levels^58^. Spearman’s *g* (derived from the Wechsler Adult Intelligence Scale (WAIS) and general cognitive ability, used in large meta-analyses, are also problematic because the first is administered as an Intelligence Quotient (IQ) test assessing verbal comprehension, perceptual reasoning, working memory, and processing speed is said to account for only up to half of the variation of cognitive function, and the latter is composed of a number of imperfectly correlated traits representing a single cognitive metric^4,7,59–62^.

This pioneer African GWAS used baseline cognitive function data from a well-characterised rural South African cohort^36^, genetic data enriched for common African variants and imputed using an African-variant-enriched reference panel, and the OCS-Plus cognitive assessment tool specifically developed for low-income settings where access to formal education is limited, and language may present barriers, to search for genetic associations with population-standardised cognitive domain scores and total cognition. Although of modest size, compared to many recent meta-analyses of cognitive traits, several genome-wide signals associated with related traits were replicated.

The genome-wide significant variant observed for association with verbal episodic memory, rs73485231, is localised to an intergenic region between *G protein subunit gamma 5* (*GNG5P5*) and *5-hydroxytryptamine receptor 2A* (*HTR2A*). Although this common variant was significantly associated with better memory performance in this sample, due to the low minor allele frequency of this SNP in other population groups, this signal was not replicated. Multiple SNPs within the same region corresponding to *GNG5P5* have been associated (although not at genome-wide significance) with gateway drug initiation in families^63^. Although the suggestive signal rs6252910 was located near *Long intergenic non-protein coding RNA 2055* (*LINC02055*) from which independent variants have been associated with self-reported mathematical ability^64^, educational attainment^65^, and the relationship between schizophrenia and cognitive function^25^ in large meta-analyses, this is insufficient to provide evidence of association. Variants mapped to the suggestively associated gene, *Beta-secretase 2* (*BACE2*), were associated with both educational attainment and mathematical ability by Lee, et al. (2018) and Okbay, et al. (2022). BACE2, although originally thought to be a *β*-amyloid precursor protein (APP)-cleaving enzyme, cleaves APP at three sites, thereby inhibiting *β*-amyloid production as well as actively degrading it^41–43^. Its overexpression in cultured cells was found to significantly lower the concentration of intracellular *β*-amyloid, and it has been hypothesised that it may influence susceptibility to AD^41–43^. The second suggestively associated gene, *transient receptor potential cation channel subfamily M member 7* (*TRPM7*), encodes a protein that has both ion channel and kinase domains that may play a role in magnesium homoeostasis^39,40^. It plays an essential role in embryogenesis and complete knockout is lethal in murine models^39,66^. Studies in *Xenopus* have shown that it is involved in neural tube closure and deficits result in a range of neural tube defects^39,66^. We replicated four reported suggestive signals previously associated with memory phenotypes; working memory, and memory performance. The replicated signal with the lowest reported *p* value, rs8067235, was the focus of a study combining computational modelling, GWAS data, and neuroimaging to validate the association of brain-specific angiogenesis inhibitor 1-associated protein 2 (*BAIAP2*) with verbal memory tasks^56^. Utilising functional MRI, they observed differences in mRNA expression between the anterior and posterior of the medial temporal lobe (the part of the brain responsible for encoding, memory storage, and recall)^67^, specifically when comparing recall of negative versus neutral memory tasks^56^. The remaining replicated signals were reported by Donati, et al. (2019) in a study looking at the overlap between measures of latent cognitive function and education in adolescents^55^.

Our suggestive signal associated with language is located within an intron of *Pleckstrin homology and RhoGEF domain* (*PLEKHG1*). Although, previous associations for this African-specific variant had not been reported for language or any other cognitive performance phenotypes, other variants within *PLEKHG1* have been associated with cerebral white matter intensities (an indication of susceptibility to vascular dementia) in Europeans and systolic blood pressure in sickle cell populations^44–46^. Suggestive signals associated with language ability were replicated, with two SNPs (in Supplementary Data 2) reported in a Danish family study assuming that receptive language in children is subject to a parent-of-origin effect^68^. The genes FUMA suggested were associated with language code for large and small mammalian mitochondrial ribosomal subunits, respectively. The association of *MRPL46* with depressive disorders was observed by Howard, et al. (2018) and Yao, et al. (2021) in their studies assessing multiple neuropsychiatric phenotypes and the possible genetic overlap between them^49,50^.

The GWAS results for executive function yielded a genome-wide significant replication of rs139493 associated with a trail-making test in 78,547 UK Biobank donors^62^. Our suggestive signal tagged *Bridging Integrator 1* (*BIN1*) has been repeatedly reported as a significant AD locus^52–54^. Although the exact mechanism is unclear, there is evidence that there are numerous ways in which *BIN1* expression may alter brain pathology^54^. BIN1 binds Tau proteins and its overexpression is correlated with AD pathology, possibly through increasing Tau production by stimulating its release from microglial cells^52–54^. In a study using transgenic mice, deposits of insoluble BIN1 were reported to accumulate alongside β-amyloid plaques in the brains of AD mice^53^. Furthermore, in knockout experiments, deficits appeared to cause impairment in spatial recognition and memory^69^.

We replicated a suggestive signal previously reported for association with visuospatial tasks in a Chinese population^70^. The most interesting gene-based result was for *limb development membrane protein 1 domain containing 2* (*LMBRD2*). Malhotra, et al. (2020) reported novel missense variants at this locus in ten individuals, each exhibiting traits which are indicative of neurodevelopmental abnormalities^71^. These included motor and intellectual delay, as well as structural abnormalities^71^.

By using window-based replication, we replicated several genome-wide significant signals reported by Davies, et al. (2018) in a study of over 300,000 individuals assessed for general cognitive function^6^. This includes signals mapped to *RNA Binding Fox-1 Homologue 1* (*RBFOX1*), a homologue to one of the suggestive gene-based association outputs from FUMA, and loci associated with various neurological disorders^6^. The SNP with the lowest *p* value for replication was rs11210871, which along with rs11577684, corresponds to loci on chromosome 1, which have been previously associated with intellectual disability and AD^6^. Loss of function variants and CNV in proximal gene *GATA zinc finger domain-containing 2B* (*GATAD2B*) have been associated with cases of intellectual disability^72,73^. Our lead SNP is a rare African-specific variant to which *RNA 7SL cytoplasmic 831 pseudogene* (*RN7SL831P*) is the closest gene. Aside from appearing in studies for educational attainment^65^ and mathematical ability^64^, single SNPs in the intergenic regions have been listed as associated with genome-wide significance to sleep-related phenotypes^74–76^ and neuropsychiatric traits like attention deficit hyperactivity disorder (ADHD)^77,78^, bipolar disorder^79^, eating disorders, and substance use^77,80,81^. Gene-based analysis suggested that *RNA Binding Fox-1 Homologue 3* (*RBFOX3*) and *mono-ADP ribosylhydrolase 2* (*MACROD2*) were associated with total cognition score. RBFOX3 is an alternative splicing regulator expressed in neurons and is a biomarker for neuron maturity^82–84^. Studies in mice and rats have elucidated its involvement in neuronal differentiation, neuro and synaptogenesis, and neurological disorders characteristic of hippocampal dysfunction^82–84^. Rare microdeletions in this gene have been found in patients suffering from childhood idiopathic epilepsy presenting with or without seizures^85^. Alterations in *RBFOX3* have been associated with specific cases of developmental delay in humans^86^ and impaired visual learning in knockout mice^82^. *RBFOX3* is expressed in neurons through all developmental stages and has been shown to interact with binding sites outside of the other RBFOX proteins^83^. Thus, it has also been suggested to play a role in miRNA biogenesis (94). Immunohistochemistry of *MACROD2* expression suggests that it may be involved in different stages of cortical neuron development and affect synaptic function^87^. Rare and de novo CNV within this gene have been observed in ADHD patients^88^. Knockout mice exhibited hyperactivity which increased with age despite slower observed movement and unusual sleep patterns similar to that seen in ADHD^89^. The most reported SNP for this locus, rs4141463, reached genome-wide significance for association with autism spectrum disorder (ASD) in a European study but was neither replicated in a later European study, nor in a study of Han Chinese^90–92^.

We observed overlapping suggestive signals for the highly correlated traits of language, executive function, and visuospatial ability on chromosomes 6 and 3. This was expected as in early childhood, executive function and language are intertwined as children with higher executive function tend to have better language skills^93^. In children with language impairments, lower executive function and attention reduced the ease at which visuospatial tasks were completed^94^. In the elderly, higher levels of education improved performance on verbal and non-verbal tasks requiring complex executive function^95^.

The adaptation of the US HRS cognition battery^96^ proved adequate in our study as a robust assessment of total cognition based on memory and orientation. Although this test originally included questions on numeracy, these were excluded as they were shown to be biased toward participants with higher levels of education^96^. The widespread use of cognitive screening tests derived from MMSEs provided a number of study phenotypes which were similar to the total cognition score as we calculated it. Using the highest level of education attained as a covariate allowed us to observe similar signals to those in large meta-analyses where educational attainment was used as a proxy for intelligence. On their own, our reported signals and the ones we replicated do not contribute to the overall heritability estimates for these phenotypes in a significant way, but there are some highly conserved loci which appear to contribute to the polygenicity of cognitive function. The OCS-Plus was a valuable tool in our study community which is known to have long-standing poor access to and quality of education, further limited by low employment rates^97^. We captured intra-population domain-specific cognition, rather than exploring the genetic basis of educational attainment as a proxy for cognitive function, as many other studies have done. Educational attainment is known to be a biased and inadequate metric in communities such as the one targeted in our research, where low levels of education observed likely correspond to extreme educational inequality in rural communities in South Africa during the apartheid era, when these individuals were young^20,31,96,97^. Having a set of well-defined traits that are population-standardised provides more accurate phenotype distributions for isolating variants associated with cognitive traits, as well as mitigating stigma attached to traits labelled inappropriately as intelligence. The use of traits like *g* fails to capture the variation observed in the actual trait vs that for *g* itself^62^. The age of the sample population was a strength as the literature states that the heritability of cognitive function changes across the lifespan and that trends between domains differ progressively with age, but stabilise at older ages^4,11^. Despite being limited by sample size, this study replicated previous genome-wide significant signals using sliding windows mostly based on studies that were performed in populations with European ancestry, informing the need for larger African cohorts where genomic and cognitive data have been collected.

The AWI-Gen/HAALSI collaboration is a trailblazer for genetic studies on neurocognitive traits in South and sub-Saharan Africa with evidence of novel associations and replication of previous associations. Larger continental African cohorts with genomic and cognitive screening data would increase the power to detect and replicate findings in other population studies, as well as provide an African cohort to use for replication of our work. Additionally, functional magnetic resonance imaging (MRI) results from this same cohort could be used to find signals linked to specific biological pathways or regions of the brain. Incorporating the OCS-Plus in future African studies may serve to establish usable datasets for monitoring cognitive health in Africa at this stage of rapid health and social transition. The generation of genomic data alongside such data will contribute to a greater understanding of how variation in African populations influences cognitive function.

## Methods

### Participants

Participants were enrolled in both the AWI-Gen and HAALSI studies. Ethical approval was granted through the University of the Witwatersrand, Johannesburg, Human Research Ethics Committee under the following certificate numbers: AWI-Gen M121029 and M170880; HAALSI M141159; and the current study M170916. Socio-demographic data, infection history, and cognitive performance data were collected from 5059 consented participants (male (*n* = 2345) and female (*n* = 2714)) aged 40 years and older recruited from Bushbuckridge, Mpumalanga (November 2014 to November 2015) and a sub-set of 2246 of these participants (male (*n* = 935) and female (*n* = 1311)) had genotype data. All participants provided written informed consent. Descriptive statistics was performed using R (R Core Team. 2020. R: A language and environment for statistical computing. R Foundation for Statistical Computing. Vienna. Austria. https://www.R-project.org/).

### Questionnaire-based cognitive assessment

The United States Health and Retirement Study (US HRS) cognition screening tool was culturally adapted and translated into the local vernacular Shangaan (also referred to as Xitsonga). This tool consisted of questions representing the domains of memory and orientation, and was scored from 0–24^31,36,96^.

### Tablet-based cognitive assessment

The Oxford Cognition Screen Plus (OCS-Plus) is an electronic cognitive assessment administered using a tablet and was validated for use in this cohort^20^. It consists of nine domain-specific cognitive tests which assess language, episodic memory, executive function, attention, and pattern recognition^20^. A factor score was derived for each cognitive domain (episodic memory, executive function, language, and visuospatial ability)^31^. This method is based on Seidlecki, Honig, and Stern (2008), and produces population-standardised domain *z*-scores for each participant^31^.

### Genotyping and imputation

Genotyping of the full AWI-Gen dataset (10,900 participants) was performed using the H3Africa array by Illumina (San Diego, CA, USA). This custom array of ~2.3 million SNPs was developed to be enriched for common African variants (http://chipinfo.h3abionet.org)^98^. Data from AWI-Gen were processed through the H3A GWAS pipeline (https://github.com/h3abionet/h3agwas), where individuals with SNP missingness greater than 0.05 were removed from the dataset^99,100^. SNPs were removed if they had genotype missingness above 0.05, minor allele frequency (MAF) below 0.01 and were not in Hardy–Weinberg equilibrium (HWE) *p* < 1 × 10^−6^. SNPs were matched to Genome Reference Consortium Human Genome build 37 (GRCh37) and ambiguous SNPs were removed^99,100^. The 1.71 million SNP dataset was then imputed using the African Genome Resources reference panel at the Sanger Imputation Server^98^. EAGLE2 was selected for the pre-phasing and positional Burrows–Wheeler transformation (PBWT) algorithm for imputation. Poorly imputed SNPs with info scores (generated by the Sanger Imputation Service: https://www.sanger.ac.uk/tool/sanger-imputation-service/) of less than 0.6, with MAF below 0.01 and HWE *p* value cut-off <10^−6^ were excluded, and the final dataset included ~14 million SNPs. The info score is an indicator of the certainty of imputation and is a score between 0 and 1, with scores closer to 1 being more accurately imputed. The AWI-Gen HAALSI samples were extracted from this dataset.

### Population structure and affinities

**Fig. 6:**
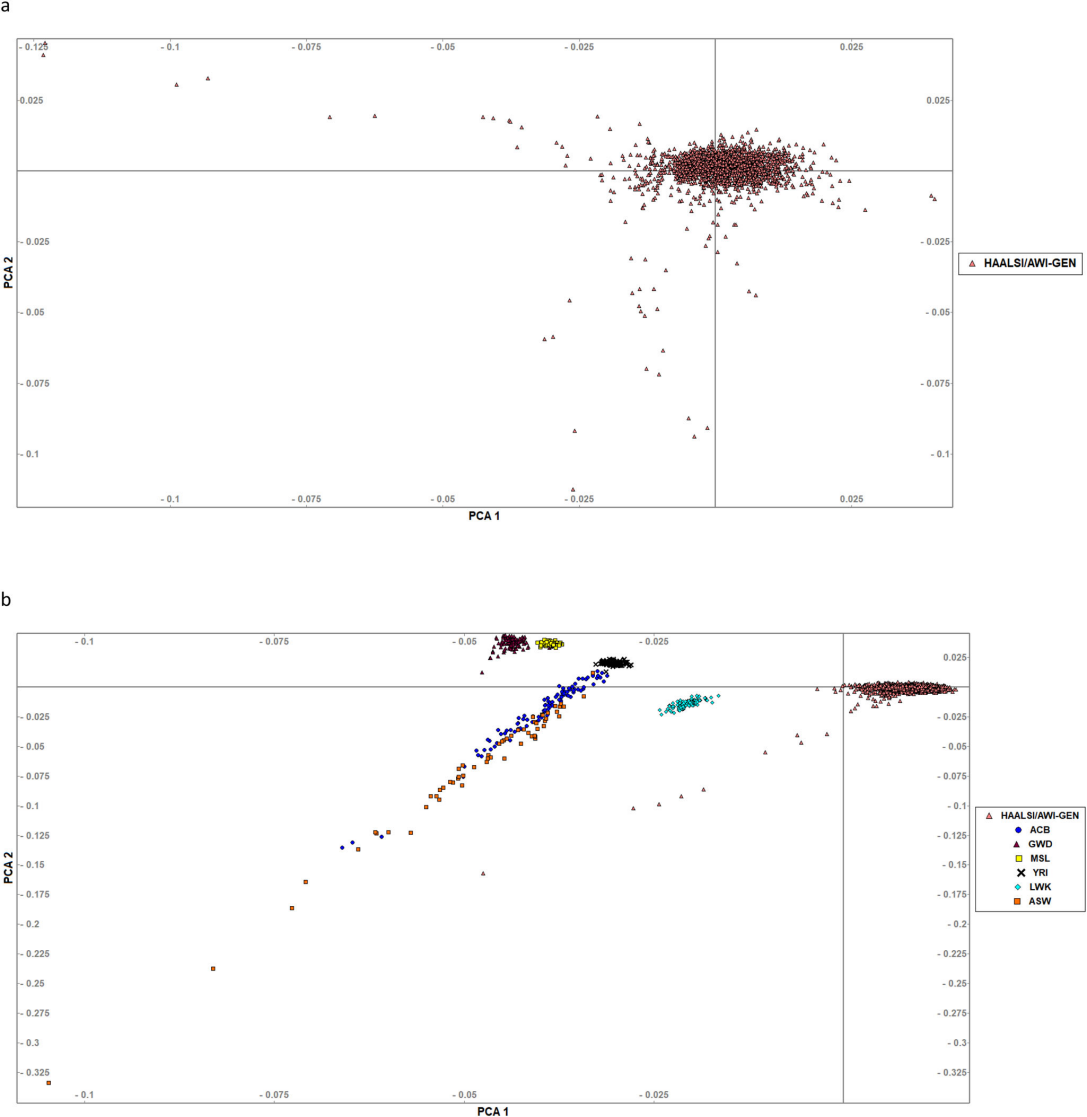
Population structure and affinities of the HAALSI/AWI-Gen participants. Principal component analysis (PCA) of individuals from the HAALSI/AWI-Gen showing PC1 and 2. **a** shows the absence of any major population structure after the removal of individuals outside of the 6 SD cut-off at five PCs. **b** shows a PCA comparison of our study participants prior to removal of outliers with African population datasets (African Caribbeans in Barbados (ACB) and Americans of African Ancestry in SW USA (ASW)), East (Luhya in Webuye, Kenya (LWK)), and West Africans (Yoruba in Ibadan, Nigeria (YRI), Gambian in Western Divisions in the Gambia (GWD), and Mende in Sierra Leone (MSL)) from the 1000 Genomes Project.

Principal component analysis (PCA) using EIGENSTRAT^101^ was performed to assess population stratification within the samples as well as to find the genetic affinities of our cohort to other African ancestry populations from the 1000 Genomes Project (1000 G Project) dataset^102^. A cut-off of ±6 standard deviations (SD) was applied to the first five PCs resulting in the removal of 35 population outliers. The sample size for further analysis was then 2211 individuals. In Fig. 6a, little evidence of population heterogeneity was shown and the PCA with other African Ancestry populations from the 1000 G Project^102^ datasets showed a distinct clustering from East, West and Central-West African populations, and African Americans (Fig. 6b).

### Statistics and reproducibility

A GWAS was performed for each of the five cognitive phenotypes. The total cognition score was captured for the entire cohort, whereas the OCS-Plus was administered to a subset of individuals. Only individuals with accompanying genomic data were included in our study sample. Total cognition was used as a continuous trait with scores ranging from 0 to 24 (*n* = 2211). Cognitive domain scores for 1887 individuals from the OCS-Plus were rank normalised using R (https://www.R-project.org/) as standardised z-scores were not normally distributed. The association was performed on the full imputed dataset using Genome-wide Efficient Mixed-Model Association (GEMMA)^103^ (https://github.com/genetics-statistics/GEMMA#gemma-genome-wide-efficient-mixed-model-association), adjusting for five PCs, age as a continuous covariate, sex, and highest level of education attained (primary, secondary, tertiary) as a categorical covariate. GEMMA was developed to perform quick association tests through univariate linear mixed models in order to correct for population substructure as well as cryptic relatedness^103^. LD scores from the 1000 G Project African reference panel and a reference panel specific to AWI-Gen’s SA data were used to adjust for LD structure^99,100^. Analyses were run on an automated H3Africa workflow for GWAS (http://github.com/h3abionet/h3agwas/)^99,100^.

### Visualisation and post-GWAS analysis

Association output files from GEMMA were analysed using Functional Mapping and Annotation of Genome-Wide Association Studies (FUMA) (https://fuma.ctglab.nl/) for partitioning of signals based on LD, visualisation and functional annotation^104^. Genome-wide significance (5 × 10^−8^) was input for analysis and the cut-off used for suggestive signals was 5 × 10^−6^. Manhattan plots and QQ plots for both SNP and gene-based association were generated using FUMA and R packages. Genomic inflation factors were calculated using a local R script. Locus zoom plots^105^ were created for selected association signals based on the summary statistics from GEMMA and SA-specific LD panel^99,100^. Kruskal–Wallis plots were constructed for comparison of cognitive function between individuals by genotype at each SNP^99,100^. GWAS Catalogue (http://ebi.ac.uk/gwas/) and Phenoscanner v2 (http://www.phenoscanner.medschl.cam.ac.uk/) were used to infer previous associations of the lead SNPs. We also studied previous associations in 100 kb genomic regions on either side of each lead SNP [accessed 10 October 2022]. Ensembl^106^ and literature mining were used to assess the functional interpretation.

### Replication

Considering the low likelihood of being able to replicate the individual genome-wide and suggestive association signals observed in our study, due to limited power and differences in LD between our study sample and European population-based cohorts, we employed a window-based approach similar to a study by Kuchenbaekar et al.^107^. Window-based replication was performed utilising add-ons from the H3A GWAS pipeline with a *P* value cut off of *p* < 1 × 10^−3^^99,100^. This cut-off was decided on the basis of empirical estimates from another study on South African populations by Mathebula, et al.^108^. Loci reported, either reaching genome-wide significance or those reported as suggestive, in previous studies of traits determined either by the similarity of methods of data collection, domain-specific tasks, and educational attainment as a proxy were prioritised for this method of replication.

### Reporting summary

Further information on research design is available in the Nature Portfolio Reporting Summary linked to this article.

## Supplementary information

Supplementary Information

Description of Additional Supplementary Files

Supplementary Data 1

Supplementary Data 2

Reporting Summary

## Data Availability

The HAALSI baseline data were publicly available at the Harvard Center for Population and Development Studies (HCPDS) programme website [www.haalsi.org]. Data were also accessible through the MRC/Wits-Agincourt Research Unit’s data repository [https://data.agincourt.co.za/index.php/catalog/18], the Inter-university Consortium for Political and Social Research (ICPSR) at the University of Michigan [www.icpsr.umich.edu] and the INDEPTH Data Repository [http://www.indepth-ishare.org/index.php/catalog/113]. Genome-wide genomic data from the AWI-Gen study are in the European Genome-phenome Archive (EGA; https://ega-archive.org/) with accession number: EGAD00010001996. The phenotype dataset is available at study number EGA00001002482 [https://ega459archive.org/datasets/EGAD00001006425]. Summary statistics for all five traits have been submitted to GWAS Catalogue under the study number GCP000532.

## Code availability

The H3A-African GWAS pipeline, QC, association testing and fine-mapping approaches are available at (https://github.com/h3abionet/h3agwas)^99,100^. Software used for analysis included PLINK 1.9 and GEMMA for GWAS analysis, EIGENSOFT and Genesis v0.2.6 for PCA analysis, R (https://www.R-project.org/)for descriptive statistics, and FUMA (fuma.ctglab.nl/fuma.ctglab.nl/) for GWAS visualisation and interpretation.
