# Supplementary material for: Genome-wide association study of population-standardised cognitive performance phenotypes in a rural South African community
Source: Commun Biol. 2023 Mar 27;6:328. doi: 10.1038/s42003-023-04636-1 (PMC10043003; doi:10.1038/s42003-023-04636-1)
Supplement: Supplementary file 1 — Supplementary Information [file 42003_2023_4636_MOESM1_ESM.pdf]

Supplementary Figure 1: Kruskal-Wallis plots showing effects of variants on cognitive traits

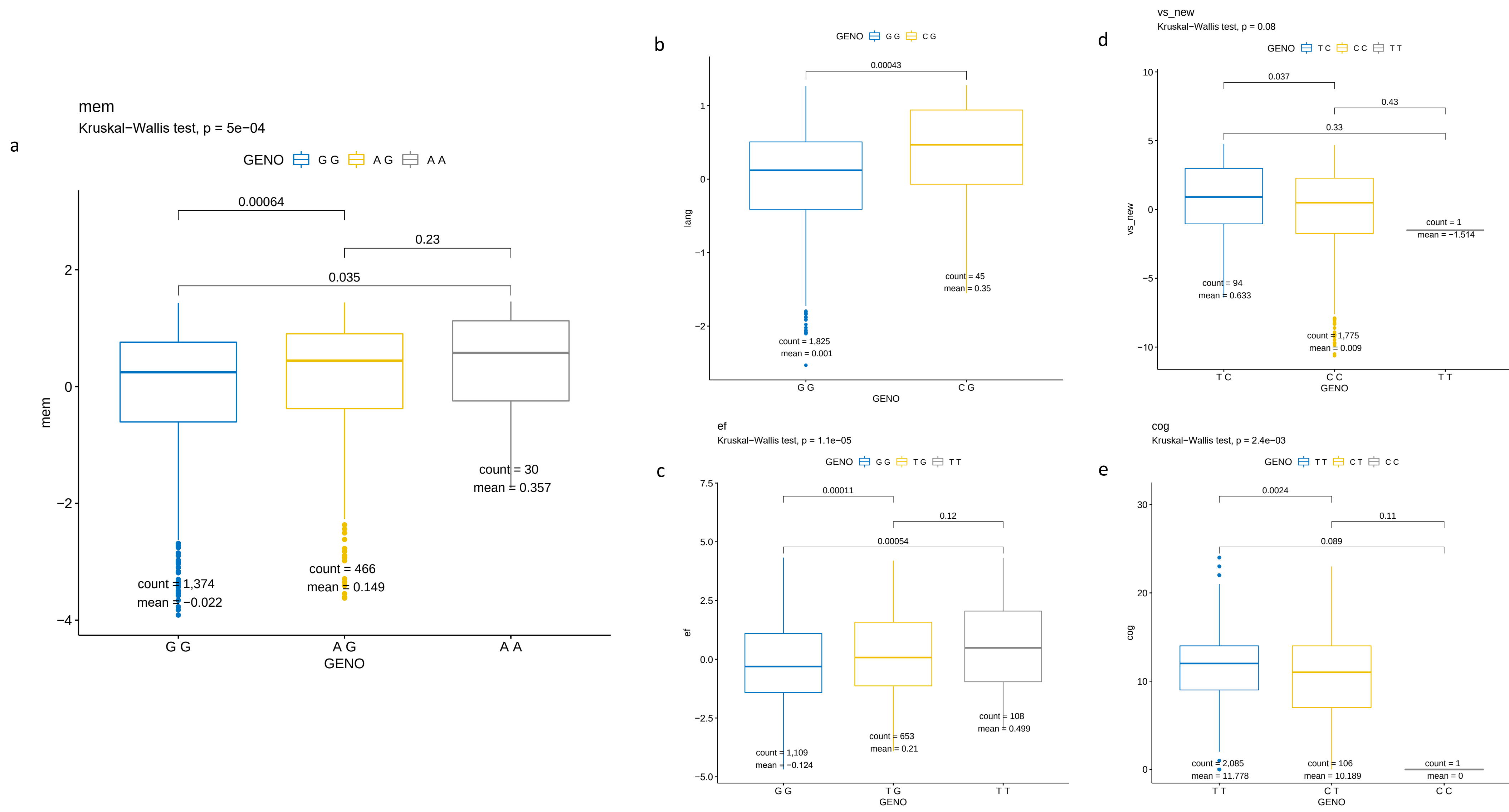

Each set of box blots represents a different phenotype by genotype plot. Each genotype is displayed in either a blue, yellow, or grey with the median line and interquartile range indicated. The whiskers above and below each box indicate Q1 and Q3 and show where samples were in the rest of the data distribution. a for rs73485231 showing difference in mean standard deviation of episodic memory between observed genotypes. b for r40578927 showing difference in mean standard deviation of language scores between observed genotypes. c for rs3845674 showing difference in mean standard deviation of executive function scores between observed genotypes. d for r91611493 showing difference in mean standard deviation of visuospatial scores between observed genotypes. e for r38832740 showing difference in mean standard deviation of total cognition scores between observed genotypes

Supplementary Table 1: FUMA gene-based associations across five cognitive traits

Study phenotype	chromosome	representative SNP	Gene	minimum GWAS p-value
Episodic memory	9	rs78943450	*TRPM6*	2.31×10^{-6}
Episodic memory	21	rs146756105	*BACE2*	3.08×10^{-6}
Language	15	rs2289416	*MRPL46*	6.24×10^{-5}
Language	15	rs2289416	*MRPS11*	3.16×10^{-6}
Visuospatial ability	4	rs112938427	*DHX15*	NA
Visuospatial ability	5	rs191611493	*LMBRD2*	1.91×10^{-6}
Visuospatial ability	5	rs193242570	*TRPC7*	2.09×10^{-4}
Visuospatial ability	7	rs4728722	*DTX2*	3.03×10^{-3}
Visuospatial ability	7	rs4728722	*UPK3B*	3.04×10^{-5}
Visuospatial ability	7	rs4728722	*POMZP3*	NA
Total cognition	17	rs9893796	RBFOX3	1.30×10^{-4}
Total cognition	20	rs6043029	MACROD2	2.70×10^{-6}
