# Supplementary material for: Genome-wide association study of population-standardised cognitive performance phenotypes in a rural South African community
Source: Commun Biol. 2023 Mar 27;6:328. doi: 10.1038/s42003-023-04636-1 (PMC10043003; doi:10.1038/s42003-023-04636-1)
Supplement: Supplementary file 2 — Description of Additional Supplementary Files [file 42003_2023_4636_MOESM2_ESM.pdf]

Description of Additional Supplementary Files

File Name: Supplementary Data 1

Description: Summary of top independent association signals with suggestive and genome-wide significance from GWAS across five cognitive phenotypes.

File Name: Supplementary Data 2

Description: Summary of GWAS replication results using exact, LD, and window-based replication (submitted as a separate xlsx. file).
