# Supplementary material for: Genome-wide association study of population-standardised cognitive performance phenotypes in a rural South African community
Source: Commun Biol. 2023 Mar 27;6:328. doi: 10.1038/s42003-023-04636-1 (PMC10043003; doi:10.1038/s42003-023-04636-1)
Supplement: Supplementary file 5 — Reporting Summary [file 42003_2023_4636_MOESM5_ESM.pdf]

nature research

Corresponding author(s): Cassandra Claire Soo
Ananyo Choudhury
Michele Ramsay

Last updated by author(s): Feb 6, 2023

Reporting Summary

Nature Research wishes to improve the reproducibility of the work that we publish. This form provides structure for consistency and transparency in reporting. For further information on Nature Research policies, see our Editorial Policies and the Editorial Policy Checklist.

Statistics

For all statistical analyses, confirm that the following items are present in the figure legend, table legend, main text, or Methods section.

n/a	Confirmed	
☐	☒	The exact sample size (*n*) for each experimental group/condition, given as a discrete number and unit of measurement
☒	☐	A statement on whether measurements were taken from distinct samples or whether the same sample was measured repeatedly
☐	☒	The statistical test(s) used AND whether they are one- or two-sided<br>*Only common tests should be described solely by name; describe more complex techniques in the Methods section.*
☐	☒	A description of all covariates tested
☐	☒	A description of any assumptions or corrections, such as tests of normality and adjustment for multiple comparisons
☐	☒	A full description of the statistical parameters including central tendency (e.g. means) or other basic estimates (e.g. regression coefficient) AND variation (e.g. standard deviation) or associated estimates of uncertainty (e.g. confidence intervals)
☐	☒	For null hypothesis testing, the test statistic (e.g. *F*, *t*, *r*) with confidence intervals, effect sizes, degrees of freedom and *P* value noted<br>*Give P values as exact values whenever suitable.*
☒	☐	For Bayesian analysis, information on the choice of priors and Markov chain Monte Carlo settings
☒	☐	For hierarchical and complex designs, identification of the appropriate level for tests and full reporting of outcomes
☐	☒	Estimates of effect sizes (e.g. Cohen's *d*, Pearson's *r*), indicating how they were calculated

Our web collection on statistics for biologists contains articles on many of the points above.

Software and code

Policy information about availability of computer code

Data collection	NA
Data analysis	PLINK 1.9, EIGENSTRAT, H3A GWAS pipeline (https://github.com/h3abionet/h3agwas), FUMA (fuma.ctglab.nl/fuma.ctglab.nl/), R (https://www.R-project.org/), GEMMA, Genesis v0.2.6 (https://www.bioinf.wits.ac.za/software/genesis/), LocusZoom (http://locuszoom.org/)

For manuscripts utilizing custom algorithms or software that are central to the research but not yet described in published literature, software must be made available to editors and reviewers. We strongly encourage code deposition in a community repository (e.g. GitHub). See the Nature Research guidelines for submitting code & software for further information.

Data

Policy information about availability of data

All manuscripts must include a data availability statement. This statement should provide the following information, where applicable:

- Accession codes, unique identifiers, or web links for publicly available datasets
- A list of figures that have associated raw data
- A description of any restrictions on data availability

The HAALSI baseline data are publicly available at the Harvard Center for Population and Development Studies (HCPDS) programme website [www.haalsi.org]. Data are also accessible through the MRC/Wits-Agincourt Research Unit's data repository [https://data.agincourt.co.za/index.php/catalog/18], the Inter-university Consortium for Political and Social Research (ICPSR) at the University of Michigan [www.icpsr.umich.edu], and the INDEPTH Data Repository [http://www.indepth-ishare.org/index.php/cata log/113]. Genome-wide genomic data from the AWI-Gen study are in the European Genome-phenome Archive (EGA; https://ega-archive.org/) with accession number: EGAD00010001996. The phenotype dataset is available at study number EGA00001002482 [https://ega459archive.org/datasets/EGAD00001006425]. The H3A-African GWAS pipeline, QC, association testing and fine-mapping approaches are available at (https://github.com/h3abionet/h3agwas) (41,42).

Field-specific reporting

Please select the one below that is the best fit for your research. If you are not sure, read the appropriate sections before making your selection.

☒ Life sciences ☐ Behavioural & social sciences ☐ Ecological, evolutionary & environmental sciences

For a reference copy of the document with all sections, see nature.com/documents/nr-reporting-summary-flat.pdf

Life sciences study design

All studies must disclose on these points even when the disclosure is negative.

Sample size	Socio-demographic data, infection history, and cognitive performance data were collected from 5,059 consented participants (male (n=2,345) and female (n=2,714)) aged 40 years and older recruited from Bushbuckridge, Mpumalanga (November 2014 to November 2015) and a sub-set of 2,246 of these participants (male (n=935) and female (n=1,311)) had genotype data. This was a cross-sectional study where sample size was determined according to data availability.
Data exclusions	Exclusion during GWAS QC was based on sexual discordance, SNP missingness, genotype missingness, minor allele frequency (MAF) and Hardy-Weinberg equilibrium, and imputation score. Further exclusion was based on data missingness in terms of covariates (age, sex, level of education) and that cognitive domain data was only available for a sub-set of the HAALSI participants. A cut-off of +/- 6 standard deviations (SD) was applied to the first 5 PCs resulting in the removal of 35 population outliers. The sample size for further analysis was then 2,211 individuals.
Replication	Window-based replication was performed utilising add-ons from the H3A GWAS pipeline with a P value cut off of p<1x10-3. This cut-off was decided on the basis of empirical estimates from another study on South African populations (Mathebula, et al. 2022). Loci reported in previous studies of traits determined either by similarity of methods of data collection, domain-specific tasks, and educational attainment as a proxy were prioritised for replication.
Randomization	NA
Blinding	NA

Reporting for specific materials, systems and methods

We require information from authors about some types of materials, experimental systems and methods used in many studies. Here, indicate whether each material, system or method listed is relevant to your study. If you are not sure if a list item applies to your research, read the appropriate section before selecting a response.

Materials & experimental systems

n/a	Involved in the study	
☒	☐	Antibodies
☒	☐	Eukaryotic cell lines
☒	☐	Palaeontology and archaeology
☒	☐	Animals and other organisms
☐	☒	Human research participants
☒	☐	Clinical data
☒	☐	Dual use research of concern

Methods

n/a	Involved in the study	
☒	☐	ChIP-seq
☒	☐	Flow cytometry
☒	☐	MRI-based neuroimaging

Human research participants

Policy information about studies involving human research participants

Population characteristics	The participants are a population cross-section with no intended enrichment for any trait/diseases. Participants (male and female) aged 40 years and over (mean=57.67 years, sd=10.93) of African descent. Cognitive trait data was collected using two screening tools - total cognition score (n=2246), cognitive domain scores (n=1903). Sociodemographic and data were captured. Whole blood samples were collected for DNA extraction and downstream analysis.
Recruitment	The Health and Aging in Africa: A Longitudinal Study of an INDEPTH Community in South Africa (HAALSI) collected baseline cognition data for over 5,059 older adults in Bushbuckridge, rural Mpumalanga, South Africa. A sub-set of 2,246 participants from this study were also recruited as part of the Africa Wits-INDEPTH Partnership for Genomic Studies (AWI-Gen) for whom genotype data were available from the Illumina Human Heredity and Health in Africa (H3Africa) array.
Ethics oversight	Ethical approval was granted through the University of the Witwatersrand, Johannesburg, Human Research Ethics Committee under the following certificate numbers: AWI-Gen M121029 and M170880; HAALSI M141159; and the study for review, M170916.

Note that full information on the approval of the study protocol must also be provided in the manuscript.
